# Effects of nine typical technologies for primary autonomous vehicles on road safety in China

**DOI:** 10.1016/j.isci.2023.106109

**Published:** 2023-02-06

**Authors:** Hong Tan, Fuquan Zhao, Zongwei Liu, Haokun Song

**Affiliations:** 1 State Key Laboratory of Automotive Safety and Energy, Tsinghua University, Beijing, China; 2 Tsinghua Automotive Strategy Research Institute, Tsinghua University, Beijing, China

**Keywords:** Safety engineering, Mechanical systems, Automation

## Abstract

The autonomous vehicle is profoundly changing the future of transportation safety. The reduction in collisions with different injury degrees and the savings of crash-related economic costs if nine autonomous vehicle technologies were promoted to be wildly available in China are evaluated. The quantitative analysis was divided into three main parts: (1) Calculate the technical effectiveness of nine autonomous vehicle technologies in collisions through a systematic literature review; (2) Apply the technical effectiveness to estimate the potential effects on avoiding collisions and saving crash-related economic costs in China if all vehicles had these technologies; and (3) Quantify the influence of current technical limitations in speed applicability, weather applicability, light applicability, and active rate on potential effects. Definitely, these technologies have different safety benefits in different countries. The framework developed and technical effectiveness calculated in this study can be applied to evaluate the safety impact of these technologies in other countries.

## Highlights

• Quantified the crash avoidance effectiveness of nine AV technologies
• Estimated potential effects on reducing collisions and related costs in China
• Quantified influence of current technical limitations on the potential effects
• The evaluation framework developed can be wildly applied to other countries

## Graphical abstract

**Figure:**
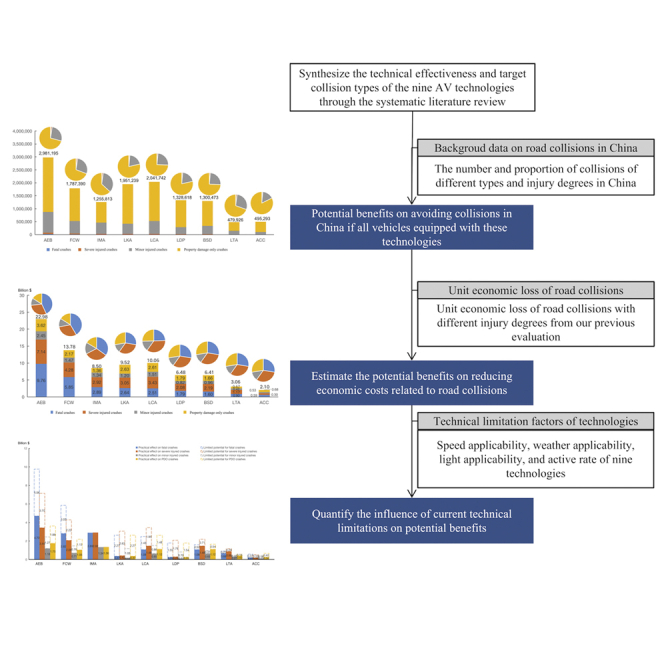

## Introduction

The road safety has gained the prominence it deserves among the world since a Decade of Action for Road Safety 2011–2020 was officially proclaimed by the United Nations General Assembly in March 2010.^1^ But at this point in 2022, most countries have not achieved the goals to halve the number of global deaths and injuries from road traffic collisions by 2020. Approximately 1.35 million people lost their lives due to road traffic collisions per year.^2^ In terms of specific countries, 62,763 people died in traffic collisions in China in 2019 and 67,759 died because of traffic collisions in 2009.^3^ The progress of traffic safety in the United States in the past ten years is similarly weak to that in China, with 33,244 fatal collisions in 2019 and 30,862 fatal collisions in 2009.^4^ Traffic collisions in various countries cause great economic costs. The annual economic cost caused by road traffic crashes is beyond imagination, as much as 0.6%–2.7% of each country’s gross domestic product.^5^^,^^6^^,^^7^^,^^8^^,^^9^^,^^10^ A new ambitious target of halving the global number of deaths and injuries from road traffic collisions by 2030 has been set in the third Global Ministerial Conference on Road Safety in 2020.^11^

Vehicle safety technology plays an important role in reducing injuries from road traffic collisions. A study in The Lancet Global Health conducted by Kavi Bhalla and Kevin Gleason assessed the reduction in deaths if vehicle safety technologies were made widely available in Latin America and the Caribbean region.^12^ Among the technologies, increasing use of seatbelts, optimization for side-impacts, and improvements to vehicle front-end design were estimated at 12.1%, 6.3%, and 6.0% fewer traffic deaths. Airbag (front and side), side door beam, and side structure and padding perform poorly in reducing traffic deaths. Brian Fildesel et al. evaluated the safety benefits of electronic stability control.^13^

The autonomous vehicle (AV) is profoundly changing the future of transportation society. By shifting responsibility for vehicle control from humans to machines, the autonomous vehicle would transform the current approach to reduce traffic fatalities from injury reduction post-collision to complete collision prevention.^14^ Many governments around the world are enthusiastic about the potential of AV technologies in reducing road traffic collisions and have already issued many policies to promote the development of the AV,^15^^,^^16^ especially in the United States, China, Japan, and the European Union. The “Automated Vehicles Comprehensive Plan” released by the US Department of transportation in 2021 explores five use cases as a way to illustrate how U.S. DOT activities address different aspects of autonomous vehicle technology, vehicles, and operational environments.^17^ In November 2020, the Ministry of Industry and Information Technology of China released “Technology Roadmap for Autonomous Vehicle 2.0”, setting the goal that the penetration rate of autonomous vehicles is expected to be 50% in 2025 and 70% in 2030.^18^ Japan and European Union also make some policies permitting autonomous vehicles to drive in the designated area.^19^

It is gradually becoming a consensus among the world’s policymakers that autonomous vehicle technologies have the potential to reduce traffic collisions and save thousands of lives every year. However, there is no consistent answer to the question of how many collisions can be prevented by AV technologies. Therefore, we aimed to evaluate the reduction in collisions and reduction in crash-related economic costs that could be achieved if autonomous vehicle technologies were wildly available in China. The SAE International classified the autonomous vehicle into level 1 to level 5 according to their level of automation, including Driver Assistance (Level 1), Partial Driving Automation (Level 2), Conditional Driving Automation (Level 3), High Driving Automation (Level 4), and Full Driving Automation (Level 5) (SAE International, 2018). The classification is similar to the standard published by the Standardization Administration of China.^20^ Driver Assistance (Level 1) and Partial Driving Automation (Level 2) are usually described as the primary autonomous vehicle, while Level 4 and Level 5 are usually called advanced autonomous vehicles. We focused on nine typical technologies for primary autonomous vehicles that have been available or will be available in the coming years for their effectiveness on traffic collisions to be systematically evaluated. These AV technologies are Forward Collision Warning (FCW), Automated Emergency Braking (AEB), Adaptive Cruise Control (ACC), Lane Departure Warning (LDW), Lane Keeping Assist (LKA), Blind Spot Detection (BSD), Lane Change Assist (LCA), Intersection Management Assist (IMA), and Left Turn Assist (LTA), as shown in the Table 1.

**Table 1:** The definition and description of the nine AV technologies

Primary AV technology	Technical description
Forward Collision Warning (FCW)	Detects a potential collision with a vehicle ahead and provides a warning to the driver
Automatic Emergency Braking (AEB)	Applies brakes automatically when a forward collision is imminent
Adaptive Cruise Control (ACC)	Automatically adjusts the vehicle’s speed to keep a distance from the vehicle in front of it
Lane Departure Warning (LDW)	Alerts the driver as the vehicle approaches or crosses lane markers
Lane Keeping Assistance (LKA)	Automatically and gently steers to prevent the vehicle from departing the lane
Blind Spot Detection (BSD)	Warns of a vehicle in the driver’s blind spot
Lane Changing Assistance (LCA)	Avoid collisions when overtaking and changing lanes
Intersection Movement Assist (IMA)	Warns drivers at intersection if another vehicle is running a red light or making a sudden turn
Left Turn Assist (LTA)	Alerts are given to the driver as they attempt an unprotected left turn across traffic

**Figure 1:**
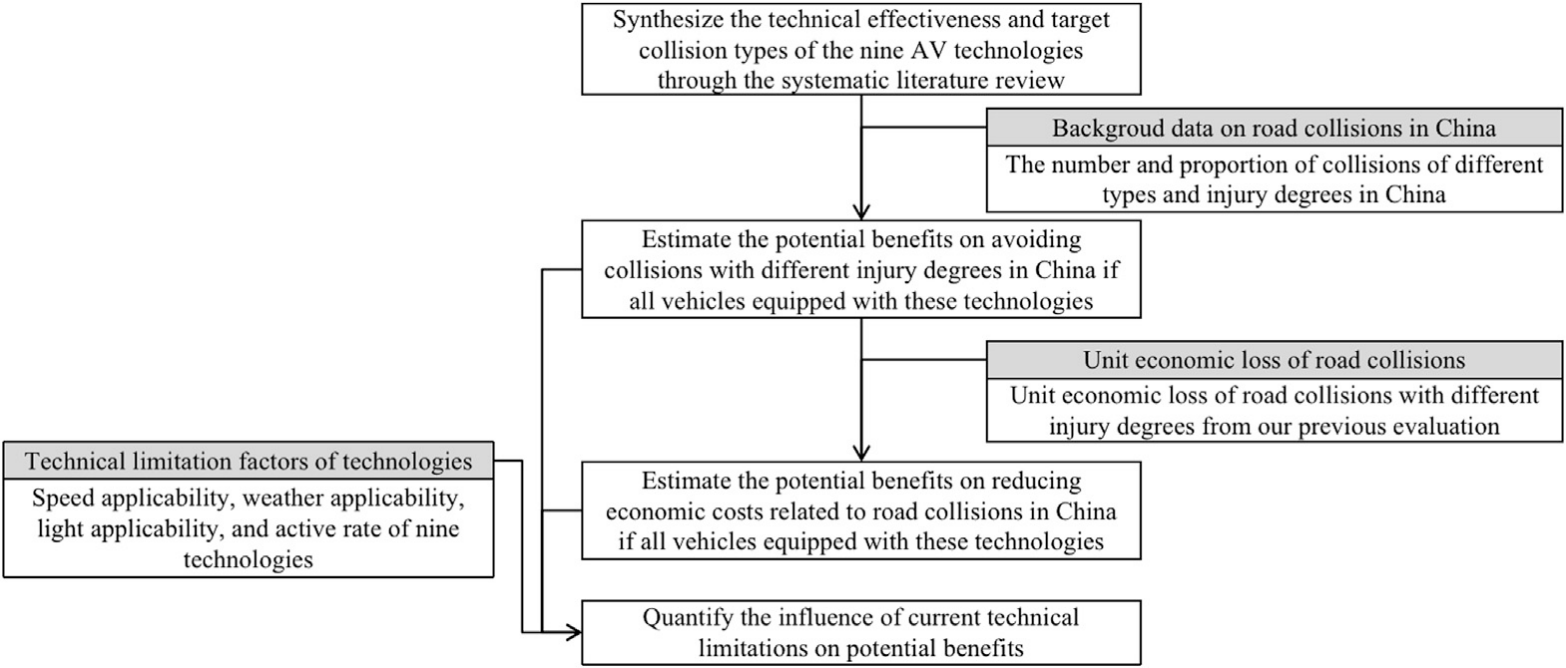
The analytical framework used in this study

The contribution of this study includes quantifying a range of safety benefits caused by the nine AV technologies, focusing on avoiding road collisions and reducing economic costs of crashes in China. Following steps are used in the framework, as shown in Figure 1: (1) Synthesize the technical effectiveness and target collision types of the nine AV technologies through the systematic literature review, (2) Estimate the potential benefits on avoiding collisions with different injury degrees and reducing crash-related economic costs in China if all vehicles had these technologies, and (3) Quantify the influence of current technical limitations on potential benefits. The framework developed and technical effectiveness calculated in this study can be applied to evaluate the safety impact of these technologies in other countries.

## Results

### Estimation of technology effectiveness

We identified target collisions types and estimated the effectiveness of nine autonomous vehicle technologies in target collisions through a systematic literature review. Three research methods are often used by scholars to evaluate the collision avoidance effectiveness of AV technologies, including Field Operation Test, Safety Impact Methodology, and Statistical Analysis Methodology.^21^ Hundreds of data on the effectiveness of nine major AV technologies are collected from hundreds of papers and reports, which constitute a relatively complete database, as shown in Table S1. The collision avoidance effectiveness of AV technologies in target collision types resulting from different studies vary greatly. It is inaccurate to rely too much on the reported effectiveness of certain research because another reported effectiveness far from it can always be found. For the effectiveness of each AV technology used for the country-level policy analysis, meta-analysis is the best way to get point effectiveness and a 95% confidence interval because lots of effectiveness results reported by lots of researchers are put together as inputs. Taking into account the sample size, and effectiveness of each study, the effectiveness obtained by meta-analysis can basically represent the true effectiveness of the technology.

According to the systematic literature review and meta-analysis, the weighted safety effectiveness and 95% confidence interval of FCW, AEB, ACC, LDW, LKA, BSD, IMA, and LTA on target collision types are evaluated to be 27.0% (95% CI: 13.8–40.1), 45.0% (37.1–53.0), 13.9% (10.1–17.6), 23.3% (17.6–29.1), 34.2% (21.5–47.0), 30.7% (21.9–39.5), 47.3% (39.7–53.9), and 50.6% (34.8–66.5) based on the selected studies from the literature. The effectiveness of LCA is currently rarely studied. So we assumed that LCA is further developed from BSD with an improvement of 57% on the effectiveness to be 48.2% (34.4–62.0), similar with FCW and LDW developed to AEB and LKA. The results of safety effectiveness calculated by meta-analysis are in a wide range. The fact is that the technical effectiveness reported by researchers all over the world are particularly different, as shown in the Table S1, which means that it will be unreliable if we only use the effectiveness from a certain research. When scientists from other countries use the weighted safety effectiveness in this paper, adding the updated technology effectiveness to the database in Table S1 to obtain more reliable effectiveness and narrower intervals is a suggested way. The target crash types of the nine AV technologies are summarized from the target crash type in Table S1, as shown in Table 2. It is not all based on assumptions but on target types mentioned in lots of literature. It could be updated as more relevant literature is published.

**Table 2:** Target collisions scenarios of the AV technologies

Collision type	FCW	AEB	ACC	LDW	LKA	BSD	LCA	IMA	LTA
Frontal collision	Y∗	Y							
Left turn into path collision (LTIP)	Y	Y						Y	Y
Right turn into path collision (RTIP)	Y	Y						Y	
Straight crossing path collision (SCP)	Y	Y						Y	
Non-intersection side collision				Y	Y	Y	Y		
Rear-end collision	Y	Y	Y						
Sideswipes collision			Y	Y	Y	Y	Y		
Collision with stationary vehicle	Y	Y	Y	Y	Y				
Other collisions involved two vehicles									
Collision with pedestrian or cyclist	Y	Y							
On-road obstacle collision	Y	Y							
Off-road obstacle collision				Y	Y				
Rollover or Falling crash									
Other single vehicle crash									

(∗Y means the type of collisions that would be effected by the AV technology).

### Potential safety benefits on avoiding collisions in China

Our findings indicate that increasing the availability of nine AV technologies will have a large effect on preventing road traffic collisions and reducing crash-related economic costs. Following results summarize the evaluated impact of nine AV technologies on collision avoiding and economic costs reducing in China.

**Figure 2:**
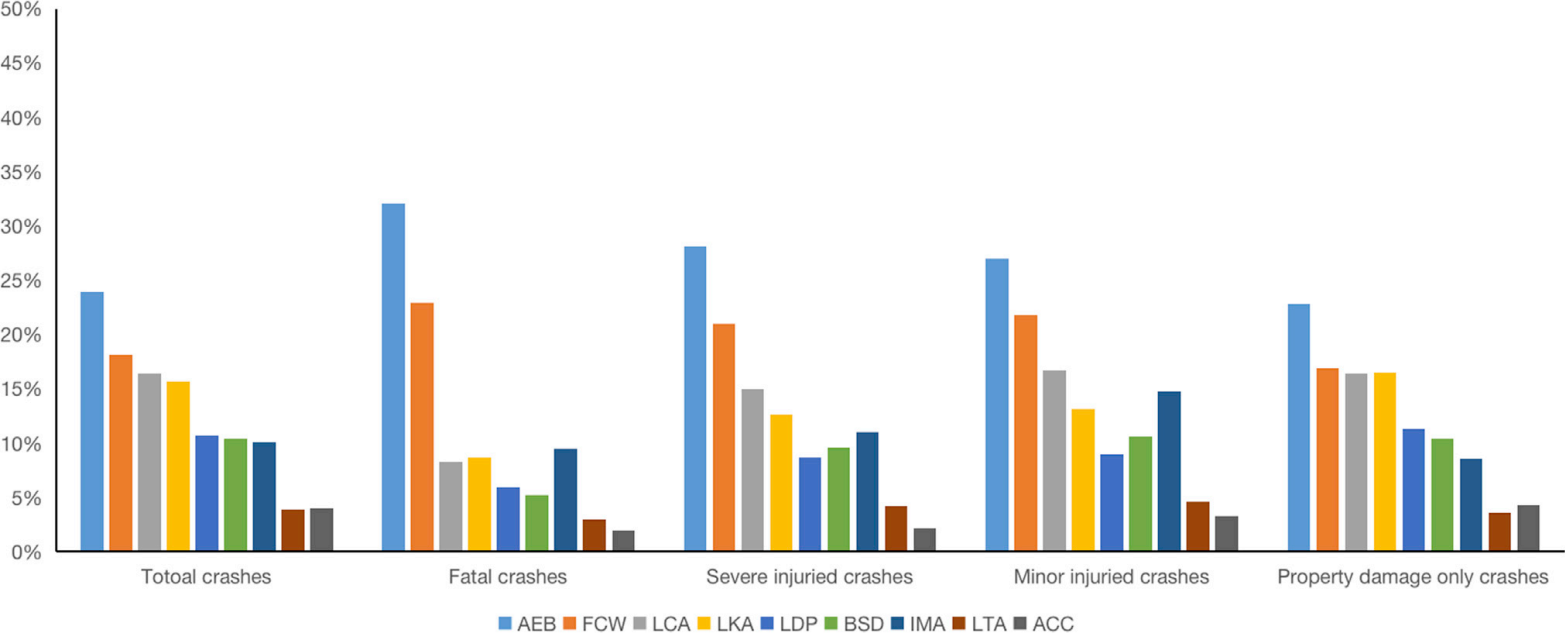
The effectiveness of nine AV technologies in reducing collisions in China (%)

**Figure 3:**
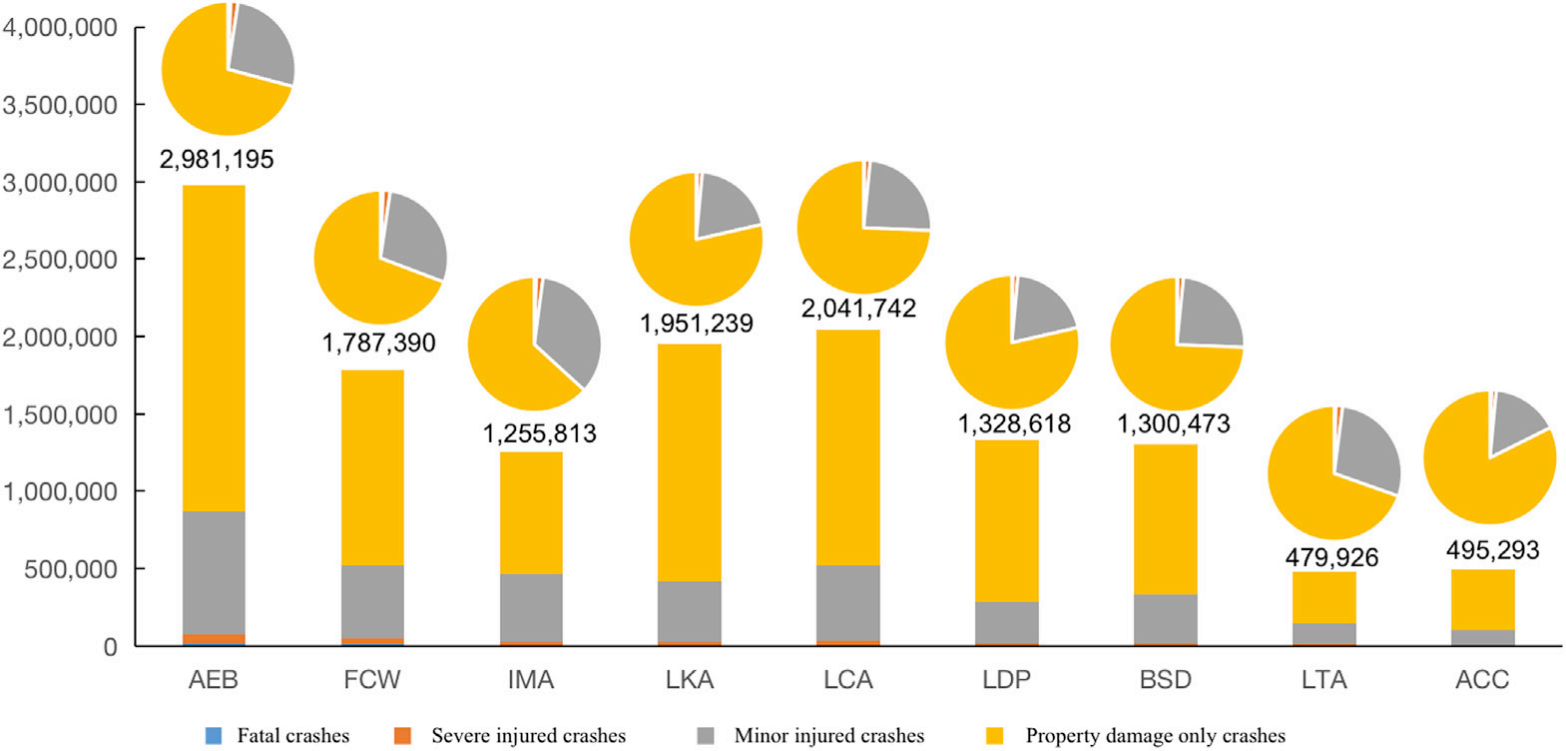
Potential benefits on avoiding collisions in China

In China, statistics on traffic collisions are reported by the Ministry of Public Security of China, which is the only official data in China. There were a total of 14.27 million road crashes in China in 2019, including 62,763 fatal crashes, 184,883 severe injured crashes, 2,953,909 minor injured crashes, and 9,271,242 property damage only (PDO) crashes.^3^ Of the nine AV technologies accessed, full penetration of AEB in the vehicle fleet would have the largest benefit for reducing collisions, resulting in 32.1% (sensitivity analysis range: 26.4–37.8) fewer fatal collisions, 29.3% (24.1–34.5) fewer severe injured collisions, 26.9% (22.2–31.7) fewer minor injured collisions, and 22.8% (18.7–26.8) fewer PDO collisions in China, as shown in Table 3 and Figure 2. The safety impact of FCW is estimated at 19.2% (9.9–28.6) fewer fatal collisions, 17.6% (9–26.1) fewer severe injured collisions, 16.2% (8.3–24) fewer minor injured collisions, and 13.7% (7–20.3) fewer PDO collisions in China. Compared with vertical technologies such as FCW and AEB, the collisions reduction in China caused by AV technologies that provide horizontal warning and control would be smaller. There would be 5.9% (4.4–7.4)–8.7% (5.4–11.9) fewer fatal collisions, 8.5% (6.4–10.6)–12.5% (7.9–17.2) fewer severe injured collisions, 9% (6.8–11.2)–13.2% (8.3–18.1) fewer minor injured collisions, and 11.3% (8.5–14)–16.5% (10.4–22.7) fewer PDO collisions if all vehicles had LDW or LKA in China. IMA is evaluated to be more beneficial to fatal collisions than LDW or LKA, with reductions in fatal collisions by 9.5% (7.9–10.8). Increasing the availability of BSD or LCA vehicles would result in 5.2% (3.7–6.8)–8.2% (5.9–10.6) fewer collisions in China. As a result, increasing penetration of ACC or LTA would have least effect, resulting in 2.0% (1.4–2.5)–3.0% (2.0–3.9) fewer deaths, 2.2% (1.6–2.8)–4.8% (3.3–6.3) fewer severe injured, 3.3% (2.4–4.1)–4.6% (3.2–6.0) fewer minor injured, and 3.6% (2.5–4.7)–4.2% (3.1–5.4) fewer PDO collisions. Besides, the nine technologies have different contributions to reducing collisions with different degrees of injury. AEB, FCW, IMA, and LKA perform better in reducing fatal collisions in China, while AEB, FCW, LKA, and LCA perform better in reducing PDO collisions. The number of crashes that could be avoided if all vehicles had these AV technologies in China is given in Table 3 and Figures 2 and 3.

**Table 3:** The effectiveness of nine AV technologies in reducing collisions in China

Estimates of collisions avoided (95% confidence interval)	Fatal collision	Severe injured collision	Minor injured collision	PDO collision
AEB	32.1% (26.4–37.8)	29.3% (24.1–34.5)	26.9% (22.2–31.7)	22.8% (18.7–26.8)
FCW	19.2% (9.9–28.6)	17.6% (9–26.1)	16.2% (8.3–24)	13.7% (7.0–20.3)
IMA	9.5% (7.9–10.8)	12.0% (10.0–13.6)	14.7% (12.3–16.8)	8.5% (7.2–9.7)
LKA	8.7% (5.4–11.9)	12.5% (7.9–17.2)	13.2% (8.3–18.1)	16.5% (10.4–22.7)
LCA	8.2% (5.9–10.6)	14.1% (10.1–18.1)	16.6% (11.9–21.4)	16.4% (11.7–21.1)
LDP	5.9% (4.4–7.4)	8.5% (6.4–10.6)	9% (6.8–11.2)	11.3% (8.5–14)
BSD	5.2% (3.7–6.8)	9.0% (6.4–11.5)	10.6% (7.6–13.6)	10.4% (7.4–13.4)
LTA	3.0% (2–3.9)	4.8% (3.3–6.3)	4.6% (3.2–6)	3.6% (2.5–4.7)
ACC	2.0% (1.4–2.5)	2.2% (1.6–2.8)	3.3% (2.4–4.1)	4.2% (3.1–5.4)

Estimates of collisions avoided	Fatal collision	Severe injured collision	Minor injured collision	PDO collision
AEB	20,142 (16,577–23,708)	54,177 (44,587–63,767)	795,917 (655,031–936,803)	2,110,959 (1,737,296–2,484,621)
FCW	12,076 (6,187–17,961)	32,482 (16,641–48,311)	477,223 (244,474–709,741)	1,265,610 (648,402–1,882,400)
IMA	5,955 (4,988–6,780)	22,128 (18,536–25,196)	435,230 (364,582–495,561)	792,501 (663,860–902,356)
LKA	5,440 (3,415–7,464)	23,173 (14,549–31,798)	389,137 (244,318–533,957)	1,533,488 (962,792–2,104,184)
LCA	5,170 (3,689–6,652)	26,052 (18,586–33,517)	491,666 (350,768–632,564)	1,518,854 (1,083,592–1,954,116)
LDP	3,704 (2,789–4,619)	15,779 (11,881–19,677)	264,967 (199,507–330,428)	1,044,167 (786,205–1,302,130)
BSD	3,293 (2,349–4,237)	16,593 (11,838–21,349)	313,163 (223,419–402,907)	967,423 (690,186–1,244,660)
LTA	1,861 (1,278–2,444)	8,872 (6,093–11,650)	136,001 (93,408–178,594)	333,193 (228,843–437,544)
ACC	1,225 (889–1,558)	4,017 (2,916–5,109)	96,141 (69,736–122,187)	393,910 (285,799–500,757)

### Potential benefits on reducing crashes-related economic costs in China

**Figure 4:**
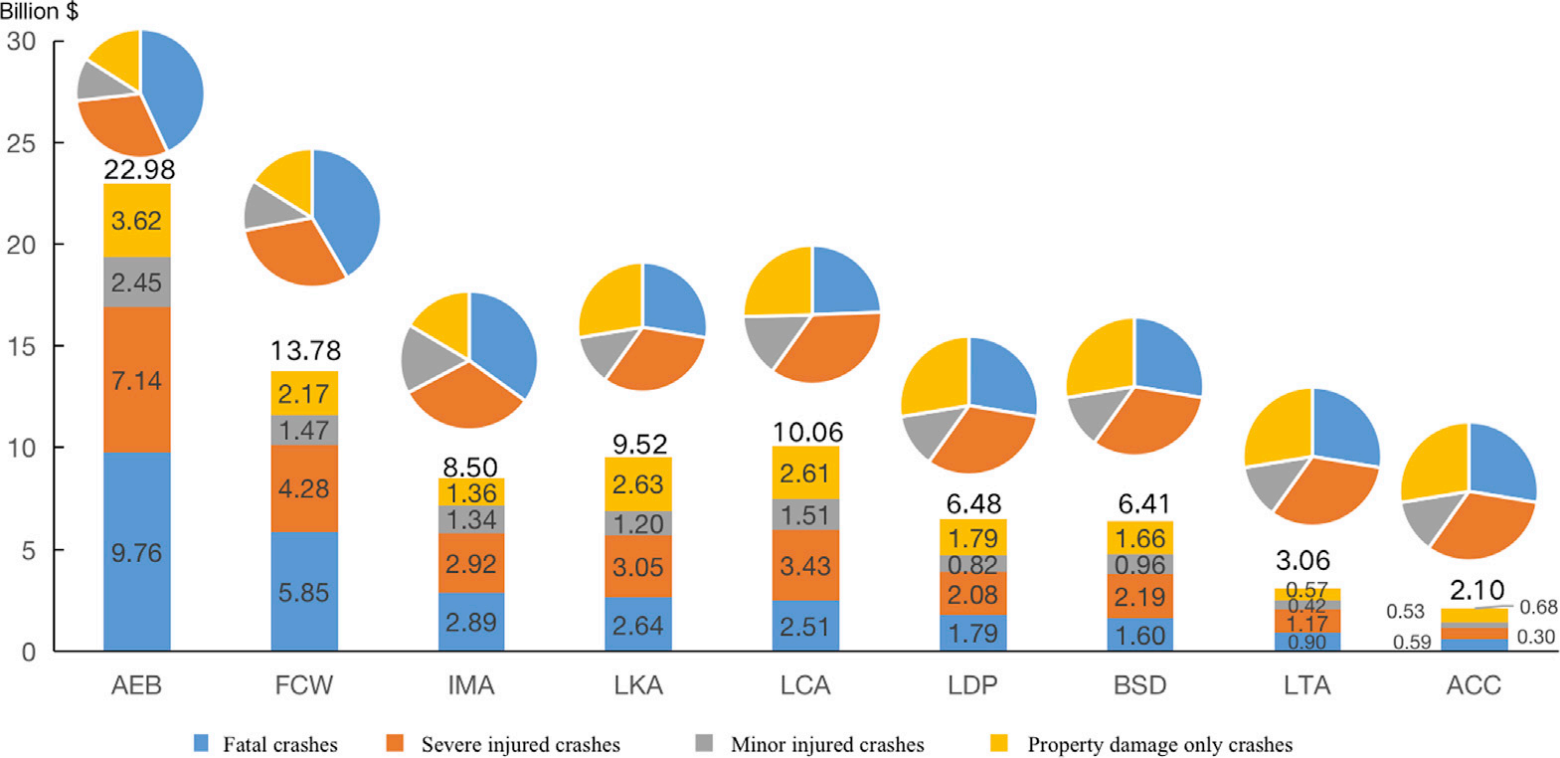
Potential benefits on reducing crash-related economic costs

Increasing the use of the nine AV technologies would have a large benefit for reducing economic costs by preventing collisions in China, as shown in Table 4 and Figure 4. Full penetration of AEB and FCW in the vehicle fleet would have the largest savings in crash-related economic costs, resulting in 22.98 (18.91–27.04) and 13.78 (7.06–20.49) billion dollars per year in China, which are far ahead of the other seven AV technologies. Increasing the availability of LCA and LKA would result in fewer 10.06 (7.18–12.94) and 9.52 (5.98–13.07) billion dollars economic losses per year. BSD and LDW, which only provide a warning when there are risks of relevant collision, are estimated at 6.41 (4.57–8.24) and 6.48 (4.88–8.09) billion dollars reduced in crash-related economic costs per year. LTA and IMA would result in 3.06 (2.10–4.02) and 8.50 (7.12–9.68) billion dollars fewer economic losses if these AV technologies were wildly available in all vehicles in China. Of the nine AV technologies assessed, ACC would have the least effect, with savings in economic costs of 2.10 (1.52–2.66) billion dollars per year in China.

**Table 4:** The impact of nine AV technologies in reducing crash-related economic costs in China

Estimates of crash-related costs reduced	Fatal collision	Severe injured collision	Minor injured collision	PDO collision	Total Savings
AEB	9.76 (8.03–11.49)	7.14 (5.88–8.41)	2.45 (2.02–2.89)	3.62 (2.98–4.27)	22.98 (18.91–27.04)
FCW	5.85 (3.00–8.70)	4.28 (2.19–6.37)	1.47 (0.75–2.19)	2.17 (1.11–3.23)	13.78 (7.06–20.49)
IMA	2.89 (2.42–3.29)	2.92 (2.44–3.32)	1.34 (1.12–1.53)	1.36 (1.14–1.55)	8.50 (7.12–9.68)
LKA	2.64 (1.65–3.62)	3.05 (1.92–4.19)	1.20 (0.75–1.64)	2.63 (1.65–3.61)	9.52 (5.98–13.07)
LCA	2.51 (1.79–3.22)	3.43 (2.45–4.42)	1.51 (1.08–1.95)	2.61 (1.86–3.36)	10.06 (7.18–12.94)
LDP	1.79 (1.35–2.24)	2.08 (1.57–2.59)	0.82 (0.61–1.02)	1.79 (1.35–2.24)	6.48 (4.88–8.09)
BSD	1.60 (1.14–2.05)	2.19 (1.56–2.81)	0.96 (0.69–1.24)	1.66 (1.19–2.14)	6.41 (4.57–8.24)
LTA	0.90 (0.62–1.18)	1.17 (0.80–1.54)	0.42 (0.29–0.55)	0.57 (0.39–0.75)	3.06 (2.10–4.02)
ACC	0.59 (0.43–0.75)	0.53 (0.38–0.67)	0.30 (0.21–0.38)	0.68 (0.49–0.86)	2.10 (1.52–2.66)

### Technical limitations of nine AV technologies

The technical limitations of these AV technologies in speed applicability, weather applicability, light applicability, and active rate deserve attention. The technical limitations are obtained from the public user manual of vehicles provided by the manufacturer and the related literature instead of the assumptions. The technical limitations in speed applicability, weather applicability, and light applicability of nine AV technologies are shown in the Table 5.

**Table 5:** Limitations of nine AV technologies

Technology	Weather applicability	Light applicability	Speed applicability
FCW	Sunny and cloudy day	Daytime and night with street light	<60 km/h (Tan et al. 2020a; i-VISTA 2018; i-VISTA 2021)
AEB	Sunny and cloudy day	Daytime and night with street light	<60 km/h (Tan et al. 2020a; i-VISTA 2018; i-VISTA 2021)
ACC	Sunny and cloudy day	Daytime and night with street light	>30 km/h (Tan et al. 2021)
LDW	Sunny and cloudy day	Daytime and night with street light	>60 km/h (Tan et al. 2020b)
LKA	Sunny and cloudy day	Daytime and night with street light	>60 km/h (Tan et al. 2020b)
BSD	Sunny and cloudy day	Daytime and night with street light	Not limited
LCA	Sunny and cloudy day	Daytime and night with street light	>15 km/h
LTA	All	Not limited	Not limited
IMA	All	Not limited	Not limited

In terms of weather applicability, light applicability, FCW, AEB, ACC, LDW, LKA, BSD, and LCA are kinds of advanced driver assistance systems. The basis of these technologies is that the object in front or side can be recognized by the camera and radar. However, the camera and radar are less effective in bad weather conditions and bad light conditions, such as sandstorms, fog, snow, and darkness.^22^ IMA and LTA are kinds of vehicle-to-vehicle (V2V) communication technologies, as shown in Table 5. For them, the object recognition is based on communication between vehicles, which could not be affected by light and weather.

In terms of speed applicability, ACC, LDW, LKA, BSD, and LCA only work when the speed is higher than the minimum active speed set by the manufacturer, the LTA and IMA works at all speeds, as shown in the Table 5. The minimum active speed for ACC, LDW/LKA, and LCA are 30, 60, and 15 km/h based on vehicles sold in China.^23^^,^^24^ FCW and AEB work efficiently only for speeds below 60 km/h. A large number of vehicle models with AEB have low test scores on 60 km/h on the AEB test conducted by i-Vista.^25^^,^^26^^,^^27^ There is no limitation on the speed of V2V technologies including LTA and IMA.

In terms of active rate factor, for FCW, LDW, and BSD, the driver may shut down the AV technologies due to the useless and presence of an uncomfortable alarm,^28^^,^^29^^,^^30^^,^^31^ as shown in the Table 6. Drivers may not be comfortable using AEB, ACC, and LKA technologies in certain contexts because the technologies perform poorly such as abrupt braking actions and unnecessary actions.^28^^,^^29^^,^^32^^,^^33^^,^^34^ Drivers who receive too many warnings or actions that they deem unnecessary or premature may grow to distrust the technology and turn it off. Activating the LCA and LTA requires turning on the turn signal when changing lanes or making left turns. The proportion of Chinese drivers turning on the turn signal when changing lanes is 65%, although the driver is required to turn on the turn signal before changing lanes.^35^ Similarly, about 75% of drivers would use the turn single when making left turns.^36^ The speed applicability factor, weather applicability factor, light applicability factor, and active rate factor of nine AV technologies were quantified, as shown in Table 6.

**Table 6:** The speed applicability factor, weather applicability factor, light applicability factor, and active rate factor of nine AV technologies

Technology	Turning on	Weather applicability	Light applicability	Speed applicability
FCW	91.5% (Flannagan et al. 2016)	89.60%	80.63%	70.45%
AEB	94.7% (Flannagan et al. 2018; Reagan et al. 2018)	89.60%	80.63%	70.45%
ACC	64.8% (SEC 2020; Pereira 2015)	89.60%	80.63%	80.85%
LDW	51.0% (Flannagan et al. 2016; Reagan et al. 2019)	89.60%	80.63%	29.55%
LKA	65.0% (Reagan et al. 2018; David et al. 2018)	89.60%	80.63%	29.55%
BSD	93.5% (Reagan et al. 2018; Braitman et al. 2010)	89.60%	80.63%	100%
LCA	65.0% (Dang 2013)	89.60%	80.63%	91.75%
LTA	75.0% (Ponziani 2012)	100%	100%	No limited
IMA	All	100%	100%	No limited

### Influence of technical limitations on potential effects

**Figure 5:**
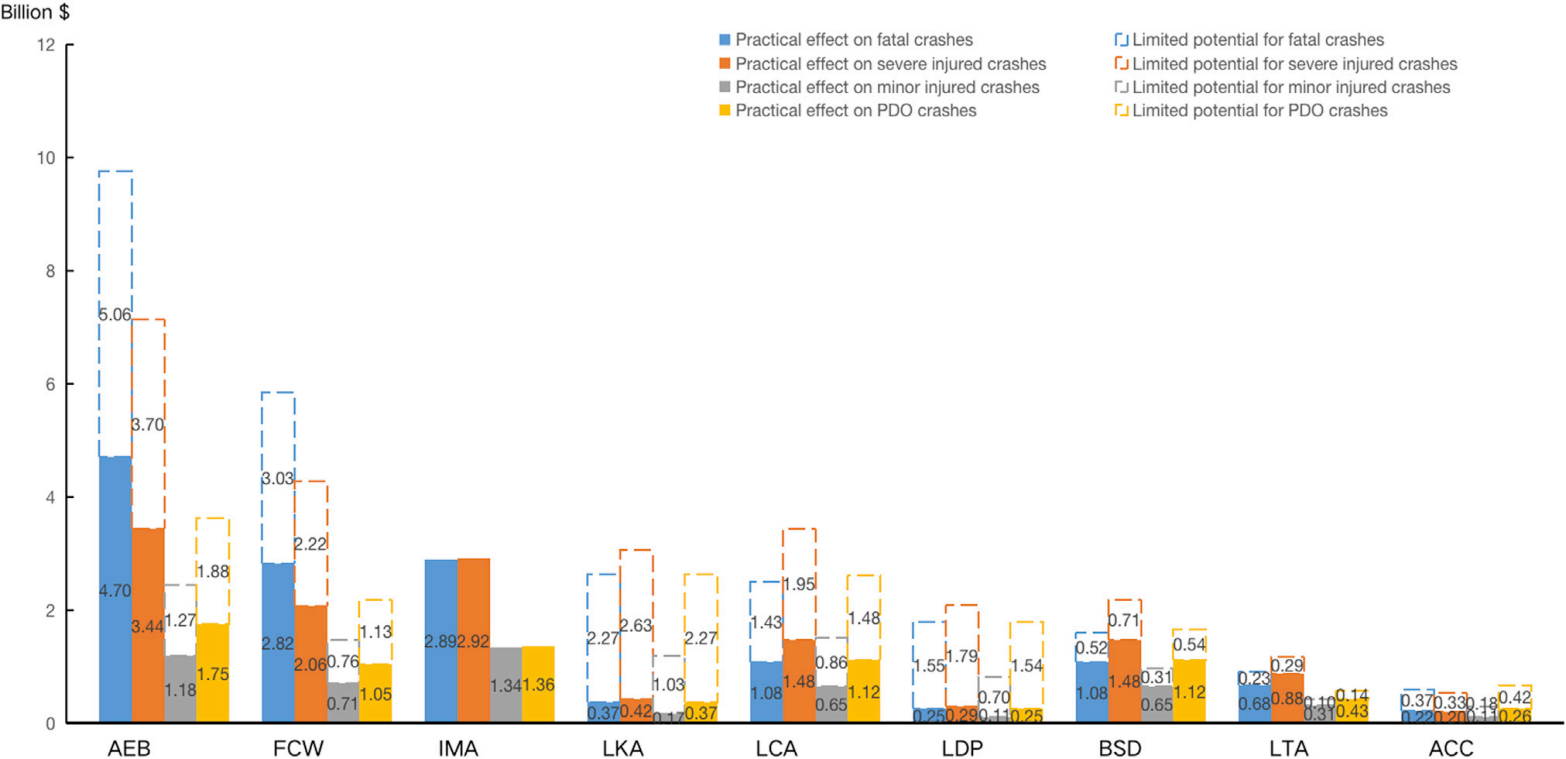
Influence of current technical limitations on potential benefits

Technical limitations make the safety effect of these technologies compromised, which is beyond the imagination. According to the result, the LDW and LKA technologies are the most affected, which only released 10.9% and 13.9% of their potential because of the limitation. The limitations have reduced the ability of LDW and LKA to avoid fatal crashes from 5.9% to 0.8% and from 8.9% to 1.2%. Meanwhile, the ACC, LCA, FCW, AEB, and BSD are affected and limited to varying degrees, with only releasing 37.8%, 43.1%, 46.5%, 48.2%, and 67.5% of their potential respectively, as shown in Figure 5. The AV technologies least affected by the limitations are LTA and IMA. 75.0% and 100% of their potential could be released. Both technologies are based on the V2V and vehicle-to-infrastructure, which make IMA and LTA work as expected at full speed range, poor lighting conditions, and bad weather conditions.

## Discussion

Our findings indicate that increasing the availability of the nine AV technologies would have a large beneficial effect on reducing road collisions and saving crash-related economic costs in China. Our analysis focuses on the nine autonomous vehicle technologies that have been available or will be available in the coming years, whose real-world effects or simulation effects have been established.

Notably, we find that the wide availability of AEB and FCW would provide the largest benefits. AEB, in particular, is one of the few AV technologies that have large benefits for frontal collision, rear-end collision, and collisions with vulnerable road users like pedestrians and cyclists, who make up a large share of road collisions in many countries, especially in low-income countries. Euro New Car Assessment Program (NCAP) has created the five-star safety rating system to help consumers identify the safest choice for their needs. Euro NCAP first started testing AEB systems in 2014 and introduced the AEB to the overall safety rating.^37^ And China NCAP has introduced the AEB to the safety rating in 2018.^38^ Ten manufacturers (Audi, BMW, Hyundai, Mazda, Mercedes-Benz, Subaru, Tesla, Toyota, Volkswagen, and Volvo) in collaboration with National Highway Traffic Safety Administration are already installing AEB in all new passenger vehicles in 2020.^39^ Forty countries led by Japan and the European Union have agreed to make AEB standard on all new cars by 2020, required by The United Nations Economic Commission for Europe.^40^ The 40 countries are only part of the world, and many more countries should make more efforts to increase the availability of AEB in their country to achieve the Sustainable Development Goal (SDG3.6).

In addition to AEB, lane keeping and lane changing systems including LDW, LKA, BSD, and LCA also have considerable effects on reducing collisions and saving costs. Compared with the focus on AEB technology, governments do not pay enough attention to these technologies at present. Similar to AEB, additional points are awarded to cars with LDW and BSD technologies in Euro NCAP, which was introduced in 2014.^37^ Unlike AEB, the BSD and LKA are scheduled to be tested as part of the overall safety rating in 2020 in China NCAP.^38^ So far, no country has issued relevant policies to make these AV technologies standards on all new passenger vehicles, even in the United States and China. Besides, IMA and LTA also deserve the government’s attention because of their contribution to avoiding traffic collisions.

Regulating and encouraging the development of autonomous vehicle technologies would have the great potential to prevent traffic collisions and reduce crash-related costs. Also, limitations of AV technologies in the range of working speed, light suitability, weather suitability, and turning on rate would have a significant impact on the safety effects. Policymakers need to work more closely with academics and manufacturers to design appropriate regulations to make sure that AV technologies themselves are safe and reliable. Some consumers are enthusiastic about using these AV technologies. Educating consumers about how these technologies work should be a concern of the manufacturers and policymakers, which could reduce the chance of these technologies being misused or shut down. Although our analysis focuses on evaluating the potential of these AV technologies, the purpose of the analysis is to encourage policy efforts that will increase the availability of these AV technologies and regulatory efforts that will improve the safety of these AV technologies.

### Limitations of the study

This research has some limitations, which can be regarded as future research topics. First, the autonomous vehicle is an integrated product that includes multiple technologies. There should be interactions between AV technologies when multiple technologies appear on one vehicle. How to quantify the effectiveness of vehicles with multiple AV technologies? The collision avoidance effectiveness of putting multiple technologies together requires and deserves more academic exploration. For some collision types, there is a coupling relationship if multiple technologies are effective. This coupling relationship is much more complex than we thought and more data are needed to develop the model. For collision types where only a single AV technology works, there is no interaction. Based on the interactions between AV technologies, the effectiveness of the vehicle with AV technologies on each collision type could be calculated. Then, considering the distribution proportion of various collision types in the country, the comprehensive collision avoidance effectiveness of the vehicle with multiple AV technologies can be estimated. Second, the market penetration rate has not been considered in this study, mainly because the relationship between penetration and safety effects has not yet been quantified by enough published academic papers. If the relationship is made into a linear relationship in this article, it may cause the evaluation to be insufficiently accurate. Third, the research did not compare the result in China with other countries. There is no doubt that the safety benefits of the nine AV technologies on avoiding crashes and reducing crash-related economic costs between China and other countries must be different. This article can provide tools and frameworks for evaluating the safety benefits of applying and promoting AV technology in other countries.

## STAR★Methods

### Key resources table

**Table:** 

REAGENT or RESOURCE	SOURCE	IDENTIFIER
**Deposited data**		

Technology effectiveness of AV technologies	Table S1	
Road safety data in China	Tables S2 and S3	

**Software and algorithms**		

Excel	N/A	N/A

### Resource availability

#### Lead contact

Further information and requests for resources and reagents should be directed to and will be fulfilled by the lead contact, Zongwei Liu (liuzongwei@tsinghua.edu.cn)

#### Materials availability

This study did not generate new materials.

### Method details

#### Method framework

Following steps are used in the framework: (1) Synthesize the technical effectiveness and target collision types of the nine AV technologies through the systematic literature review, (2) Estimate the potential benefits on avoiding collisions with different injury degrees and reducing crash related economic costs in China if if all vehicles had these technologies, (3) Quantify the influence of current technical limitations on potential benefits.

(Equation 1) $${PCR}_{i,k}=\sum_{j=1}^{14}{{CP}_{j,k}*TEF}_{i,j}*{\bar{{Eff}_{i}}}^{*}$$

(Equation 2) $${NCR}_{i}=\sum_{k=0}^{3}\sum_{j=1}^{14}{CN}_{k}*{{CP}_{j,k}*TEF}_{i,j}*{\bar{{Eff}_{i}}}^{*}$$

(Equation 3) $${PELS}_{i}=\sum_{k=0}^{3}\sum_{j=1}^{14}{CN}_{k}*{{CP}_{j,k}*TEF}_{i,j}*{\bar{{Eff}_{i}}}^{*}*{UEL}_{k}$$

(Equation 4) $${LELS}_{i}=\sum_{k=0}^{3}\sum_{j=1}^{14}{CN}_{k}*{{CP}_{j,k}*TEF}_{i,j}*{\bar{{Eff}_{i}}}^{*}*{LF}_{i}*{UEL}_{k}$$

(Equation 5) $${LF}_{i}={AR}_{i}*{WLF}_{i}*{LLF}_{i}*{SLF}_{i}$$

Equations 1, 2, 3, 4, and 5 present the major calculation flows embedded in the model to evaluate the safety impact of each technologies. Technical effectiveness and target collision types of the nine AV technologies, the number and proportion of collisions of different types and injury degrees in China, unit economic loss of road collisions with different injury degrees from our previous evaluation, technical limitations on speed applicability, weather applicability, light applicability, and active rate of nine technologies are considered in the evaluation.Where, ${PCA}_{i,k}$ is the potential percentage of crashes reduction per year by i AV technology, in injured degree k; ${NCR}_{i}$ is the potential number of crashes reduction per year by i AV technology; ${PELS}_{i}$ is the potential economic loss saving per year by i AV technology; ${LELS}_{i}$ is the limited economic loss saving per year by i AV technology; ${CN}_{k}$ is the crash number of injured degree k; ${CP}_{j,k}$ is the proportion of collision type j, in injured degree k; ${TEF}_{i,j}$ is the technology effect factor of i AV technology on collision type j; ${\bar{{Eff}_{i,j}}}^{*}$ is the fixed weighted mean effectiveness of i AV technology in collision type j; ${UEL}_{k}$ is the unit economic loss of injured degree k; ${LF}_{i}$ is the limitation factor of i AV technology; ${TLF}_{i}$ is the active rate of i AV technology; ${WLF}_{i}$ is the weather applicability factor of i AV technology; ${LLF}_{i}$ is the light applicability factor of i AV technology; ${SLF}_{i}$ is the speed applicability factor of i AV technology;

#### Estimation of technology effectiveness

Hundreds of data on the effectiveness of nine major AV technologies are collected from hundreds of papers and reports, which constitutes a relatively complete database, as shown in Table S1. Most studies give a range of effectiveness, and the range is averaged to get the crash avoidance effectiveness of each study for Table S1.

In the meta-analysis, the statistical weight assigned to each study was calculated by the random effects model to make sure that the assessment results would be closer to the true effectiveness.^41^^,^^42^^,^^43^ This database would provide key support for similar research on the safety impacts of AV technologies on road safety in other countries.

The meta-analysis was conducted to evaluate the safety effectiveness of each AV technology based on the data in the Table S1. For a given AV technology, Equations 6 and 7 present the major calculation flows to evaluate the weighted mean effectiveness and the 95% confidence interval for the weighted mean effectiveness based on a set of estimates. Equations 8, 9, 10, 11, 12, and 13 provide the details on all variables.

(Equation 6) $${\bar{{Eff}_{i}}}^{*}=\frac{\sum_{m=1}^{g_{i}}{Eff}_{i,m}*{W_{m}}^{*}}{\sum_{m=1}^{g_{i}}{W_{m}}^{*}}$$

(Equation 7) $$95\%CI={\bar{{Eff}_{i}}}^{*}\pm 1.96*\frac{1}{\sqrt{\sum_{m=1}^{g_{i}}{W_{m}}^{*}}}$$

(Equation 8) $${W_{m}}^{*}=\frac{1}{{{se}_{m}}^{2}+{\sigma_{\theta }}^{2}}$$

(Equation 9) $${{se}_{m}}^{2}=\frac{{Eff}_{i,m}*\left({1-Eff}_{i,m}\right)}{n-1}$$

(Equation 10) $${\sigma_{\theta }}^{2}=\frac{Q-g+1}{\sum W_{m}-\left(\sum {W_{m}}^{2}/\sum W_{m}\right)}$$

(Equation 11) $$Q=\sum_{m}W_{m}*{\left(\bar{{Eff}_{i}}-{Eff}_{i,m}\right)}^{2}$$

(Equation 12) $$\bar{{Eff}_{i}}=\frac{\sum_{m=1}^{g_{i}}{Eff}_{i,m}*W_{m}}{\sum_{m=1}^{g_{i}}W_{m}}$$

(Equation 13) $$W_{m}=\frac{1}{{{se}_{m}}^{2}}$$

Where, ${\bar{{Eff}_{i}}}^{*}$ is the fixed weighted mean effectiveness of i AV technology; 95%CI is the 95% confidence interval for the weighted mean effectiveness; ${W_{m}}^{*}$ is the fixed statistical weight of the study m; ${se}_{m}$ is the SE of the study m; N is the sample size of each study; ${Eff}_{i,m}$ is the effectiveness of i AV technology, in study m; ${\sigma_{\theta }}^{2}$ is the between study variance; Q is the homogeneity test statistic; $W_{m}$ is the initial statistical weight assigned to each study; $\bar{{Eff}_{i}}$ is the initial weighted mean effectiveness of i AV technology.

In terms of ${TEF}_{i,j}$, if the technology i could not effect the collision type j, the technology effect factor is zero, otherwise the technology effect factor is 1, as shown in Table 2.

#### Background data on road collisions in China

The target collision types that can be avoided by different AV technologies are different. The collision avoidance effectiveness of different AV technology is also different. To identity the number and proportion of collisions with different types and different injury degrees, we obtained related data from the Annual Report on Road Traffic Accidents of China.^3^ When scientists from other countries use the framework and data in this paper, they can replace the collision data with their country’s collision data. The types of collisions were grouped into 14 groups((eg, sideswipe collisions, rear-end collisions, and frontal collisions). The detailed distribution of different collision types in China’s road traffic crashes is shown in Table S2. And the injury degrees were grouped into four groups for economic impact evaluation: fatal collisions, severe injured collisions, and minor injured collisions, and property damage only collisions.

#### Unit economic loss of road collisions in China

The annual comprehensive economic losses of road crash in the United States, United Kingdom, Sweden, Germany, Australia and India have been estimated by scholars around the world.^5^^,^^6^^,^^7^^,^^8^^,^^9^ But this issue has not been evaluated in China. Considering the economic losses of property damage, productivity loss, medical cost, travel delay time cost, legal cost, and insurance cost, we proposed a comprehensive model to evaluate the economic loss of road traffic crashes in China based on the data obtained from official Chinese agencies. The total economic losses of traffic collisions in China were estimated to be 72.6 billion USD in 2017, which was equivalent to 0.60% of the GDP of China.^44^ The overall economic losses caused by road traffic collisions are beyond imagination. The more serious the injury, the higher the unit economic loss. The unit economic loss of a PDO collision, minor injured collision, severe injured collision and fatal collision was estimated to be 1,670, 2,995, 128,195, and 471,192 dollars in 2017 respectively. More details in economic losses of road crashes could be found in our previous published paper.^44^

#### Technical limitation factors

The technical limitations in speed applicability, weather applicability, light applicability and active rate of nine AV technologies could be found in the Tables 5 and 6, which are obtained from the public user manual of vehicles provided by the manufacturer and the related literature instead of the assumptions.

The weather applicability factor, light applicability factor, speed applicability factor is calculated base on the limitations in Table 5 and corresponding distribution in China crashes in 2019,^3^ as shown in Table S3. For speed information, only the gears used by the vehicle when crashes occurred are counted in the accident statistics report,^3^ as shown in Table S3. When calculating speed applicability factor, there is a corresponding relationship between gear and speed range.^45^ If the gear is the first gear, the speed range is assumed to be from 0 to 15 km/h, the second gear corresponds to 15–35 km/h, the third gear corresponds to 35–50 km/h, the fourth gear corresponds to 50–60 km/h, and a gear higher than the fourth gear corresponds to a speed greater than 60 km/h. Meanwhile, neutral gear, unclear, and automatic transmission are excluded since there is no speed information for automatic transmission.

### Quantification and statistical analysis

The statistical analysis and plots were performed with Excel.

## Data Availability

All data and method details could be found at the paper and supplemental information. Any additional information required to reanalyze the data reported in this paper is available from the lead contact upon request.
