# Supplementary material for: Effects of nine typical technologies for primary autonomous vehicles on road safety in China
Source: iScience. 2023 Feb 6;26(3):106109. doi: 10.1016/j.isci.2023.106109 (PMC9984551; doi:10.1016/j.isci.2023.106109)
Supplement: Document S1. Tables S1–S3 [file mmc1.pdf]

iScience, Volume *26*

Supplemental information

Effects of nine typical technologies for primary autonomous vehicles on road safety in China

Hong Tan, Fuquan Zhao, Zongwei Liu, and Haokun Song

Supplemental information

Table S1. Evidence for the crash avoidance effectiveness of the nine AV technologies (prioritized for the technology, method, crash type, and date of source). *Related to STAR Methods.*

Technology	Method	Sample size	Crash type	Effectiveness.	Source
FCW-V2V	SIM	40	rear-end crash	41%	S1
FCW	SAM	4,125	rear-end crash	21%	S2
FCW	SAM	3,629	rear-end crash	21%	S3
AEB	SIM	243	pedestrian	42%	S4
AEB	SIM	1,943	pedestrian and cyclists	59%	S5
AEB	SIM	338	pedestrian	60%	S6
AEB	SIM	103	pedestrian, front, rear-end	18%	S7
AEB	SIM	282	rear-end crash	40%	S8
AEB	SIM	1,103	rear-end crash	72%	S9
AEB	SAM	23,649	rear-end crash	43%	S10
AEB	SAM	-	rear-end crash	38%	S11
AEB	SAM	1,178	rear-end crash	46%	S2
AEB	SAM	454	rear-end crash	27%	S12
AEB	SAM	84,000	rear-end crash	34%	S13
AEB	SAM	1,673,000	rear-end, single crash	66%	S14
AEB	FOT	1,021	rear-end crash	45%	S15
ACC	FOT	20	rear-end crash	13%	S16
ACC	FOT	100	rear-end crash	12%	S17
ACC	SIM	5,000	rear-end crash	14%	S18
ACC+FCW	FOT	100	rear-end crash	16%	S19
ACC+AEB	SAM	35,401	rear-end crash	45%	S20
LDW	SIM	478	lane departure crash	29%	S21
LDW	SIM	478	lane departure crash	26%	S22
LDW	SIM	128	lane departure crash	27%	S23
LDW	SIM	76	lane departure crash	47%	S24
LDW	SIM	478	lane departure crash	17%	S25
LDW	SAM	5,932	sideswipe, head on, runoff road crash	48%	S24
LDW	SAM	22,65,000	sideswipe, single, front	25%	S14
LDW	SAM	5,433	sideswipe crashes, single-vehicle, and head-on	18%	S25
LDW	SAM	166,000	sideswipe crash	10%	S13
LDW	SAM	5,267	lane departure crash	10%	S2
LDW	FOT	108	lane departure crash	19%	S26
LKA	SIM	128	lane departure crash	51%	S23
LKA	SIM	478	lane departure crash	35%	S22
LKA	SAM	14,779	lane departure crash	30%	S20
LKA	SAM	2,624	lane departure crash	20%	S2
LKA	SAM	-	head-on, single crashes	32%	S27
BSD	SAM	9716	lane change crash	32%	S20
BSD	SAM	561	lane change crash	26%	S2
BSD	SAM	15507	lane change crash	14%	S28
BSD	SAM	488	lane change crash	31%	S29
BSD	SAM	4620	lane change crash	14%	S30
BSD	FOT	33	lane change crash	58%	S31
BSD	FOT	108	lane change crash	41%	S26
BSD-V2V	SIM	28	lane change crash	39%	S32
BSD-V2V	SIM	140	lane change crash	30%	S1
IMA-Warning	SIM	144	intersection crash	48%	S33
IMA-Warning	SIM	96	intersection crash	45%	S34
IMA-Warning	SIM	144	intersection crash	50%	S35

IMA-Warning	SIM	770	straight crossing paths crash(SCP)	23%	S36
IMA-Warning	SIM	459	SCP crash	35%	S37
IMA-Braking	SIM	84	SCP crash	64%	S1
IMA-Braking	SIM	459	SCP crash	49%	S37
IMA-Braking	SIM	770	SCP crash	42%	S36
IMA-Braking	SIM	792	SCP crash	67%	S38
IMA-Braking	SIM	40	intersection crash	53%	S32
LTA-Warning	SIM	96	left turn across path crash	55%	S33
LTA-Braking	SIM	96	left turn across path crash	56%	S35
LTA-Braking	SIM	501	left turn across path crash	32%	S39
LTA-Braking	SIM	501	left turn across path crash	60%	S39

Table S2: The detailed distribution of different collision types in China's road traffic crashes. *Related to STAR Methods.*

Collision in 2019[S40]	Collision type	Fatal collision	Severe injured collision	Minor injured collision	PDO collision
Collision with vehicles	Frontal collision	4,076	10,649	123,132	228,225
	Left turn into path collision (LTIP)	3,677	17,531	268,748	658,418
	Right turn into path collision(RTIP)	3,484	10,221	254,642	344,510
	straight crossing path collision (SCP)	5,419	18,996	396,070	671,299
	Non-intersection side collision	7,354	38,356	537,497	1,460,842
	Rear-end collision	5,455	13,247	209,644	1,146,752
	Sideswipes collision	3,375	15,704	482,769	1,690,960
	Collision with stationary vehicle	3,489	10,259	81,354	680,647
	Other collision with two vehicles	560	1,892	30,223	88,654
Single vehicle collision	Collision with pedestrian or cyclist	17,473	36,085	399,606	313,549
	On road obstacle collision	1,667	3,349	34,688	645,441
	Off road obstacle collision	1,667	3,349	34,688	645,441
	Rollover or Falling crash	3,881	2,880	53,181	230,832
	Other single vehicle crash	1,186	2,365	47,668	465,673
Total collisions		62,763	184,883	2,953,909	9,271,242

Table S3. The distribution of weather condition, light condition and speed condition in China's road traffic crashes. *Related to STAR Methods.*

Distribution[S40]	Category	Proportion of crashes
Weather condition distribution	Sunny day	74.48%
	Cloudy day	15.12%
	Rainy day	9.58%
	Snowy day	0.44%
	Foggy day	0.27%
	Windy day	0.02%
	Sandstorm	0.01%
	Hail day	0.00%
	Smoggy	0.01%

	Other		0.07%
Light condition distribution	Daytime		58.26%
	Night with street light		22.37%
	Night without street light		14.72%
	Dusk		2.08%
	Dawn		2.57%
Speed distribution	Manual transmission	Neutral gear	17.05%
		Gear 1	1.56%
		Gear 2	2.75%
		Gear 3	4.98%
		Gear 4	4.04%
		Gear >=5	5.02%
		Reverse Gear	0.57%
		Unclear	30.89%
	Automatic transmission	Automatic transmission	32.69%

Supporting References

[S1]. Guglielmi, J., Yanagisawa, M., Swanson, E., Stevens, S., & Najm, W. J. (2017). Safety benefits of heavy-vehicle crash warning applications based on vehicle-to-vehicle communications(Report No. DOT HS 812 429). Washington, DC: National Highway Traffic Safety Administration.

[S2]. Andrew J. Leslie, Raymond J. Kiefer, Michael R. Meitzner, Carol A. Flannagan. (2019). Analysis of the Field Effectiveness of General Motors Production Active Safety and Advanced Headlighting Systems. UMTRI-2019-6

[S3]. Hickman, J. S., Guo, F., Camden, M. C., Medina, A., Hanowski, R. J., Mabry, E. (2013). On board Safety System Effectiveness Evaluation Final Report. Report No. FMCSA-RRT-12-012. Washington, D.C.: Federal Motor Carrier Safety Administration.

[S4]. Rosén E, Källhammer J, Eriksson D, Nentwich M, Fredriksson R, Smith K. (2010). Pedestrian injury mitigation by autonomous braking. Accident analysis and prevention, 42(6), pp 1949-1957.

[S5]. Kovaceva, J., Bálint, A., Schindler, R., & Schneider, A. (2020). Safety benefit assessment of autonomous emergency braking and steering systems for the protection of cyclists and pedestrians based on a combination of computer simulation and real-world test results. Accident Analysis & Prevention, 136, 105352.

[S6]. Samantha H. Haus, Rini Sherony & Hampton C. Gabler (2019) Estimated benefit of automated emergency braking systems for vehicle–pedestrian crashes in the United States, Traffic Injury Prevention, 20:sup1, S171-S176. 10.1080/15389588.2019.1602729

[S7]. Doecke, S., Anderson, R., Mackenzie, J., & Ponte, G. (2012). The potential of autonomous emergency braking systems to mitigate passenger vehicle crashes. Policing and Education Conference. 4-6: 11 p.

[S8]. Woodrooffe, J., Blower, D., Bao, S., Bogard, S., Flannagan, C., Green, P. E., LeBlanc, D. (2012). Final Report: Performance Characterization and Safety Effectiveness Estimates of Forward

删除[H]: , November

删除[H]: “

删除[H]: ”

删除[H]: , 2013

删除[H]:

删除[H]: ‘

删除[H]: ’

设置格式[H]: 字体: 10 磅, 字体颜色: 自动设置, （中文）

删除[H]: Kovaceva J , Bálint, András, Schindler R , et al. Safety benefit assessment of autonomous emergency braking and steering systems for the protection of cyclists and pedestrians based on a combination of computer simulation and real-world test results[J]. Accident Analysis & Prevention. Volume 136, March 2020, 105352

删除[H]: , DOI:

设置格式[H]: 字体: 10 磅, 字体颜色: 自动设置, （中文）

删除[H]: Doecke, Samuel David; Anderson, Robert William Gerard; Mackenzie, James Richard Ryder; Ponte, Giulio The potential of autonomous emergency braking systems to mitigate passenger vehicle crashesProceedings of the Australasian Road Safety Research

删除[H]: ,

删除[H]: , held in Wellington, New Zealand,

删除[H]: October, 2012

删除[H]: “

Collision Avoidance and Mitigation Systems for Medium/Heavy Commercial Vehicles. Docket No. NHTSA-2013-0067. Washington, D.C.: National Highway Traffic Safety Administration.

[S9]. Georgi A, Zimmermann M, Lich T, Blank L, Kickler N, Marchthaler R. (2009). New approach of accident benefit analysis for rear end collision avoidance and mitigation systems', in Proceedings of the 21st International Technical Conference on the Enhanced Safety of Vehicles, Stuttgart.

[S10]. J.B. Cicchino. (2017). Effectiveness of forward collision warning and autonomous emergency braking systems in reducing front-to-rear crash rates. Accid. Anal. Prev., 99, pp. 142-152

[S11]. B. Fildes, M. Keall, N. Bos, A. Lie, Y. Page, C. Pastor, L. Pennisi, M. Rizzi, P. Thomas, C. Tingvall. (2015). Effectiveness of low speed autonomous emergency braking in real-world rear-end crashes. Accid. Anal. Prev., 81, pp. 24-29

[S12]. Irene Isaksson-Hellman & Magdalena Lindman. (2016). Evaluation of the crash mitigation effect of low-speed automated emergency braking systems based on insurance claims data. Traffic Injury Prevention, 17:sup1, 42-47. 10.1080/15389588.2016.1186802

[S13]. Jermakian, J. S. (,2012). Crash Avoidance Potential of Four Large Truck Technologies." Accident Analysis and Prevention 49: 338-346.

[S14]. Jermakian J S . (2011). Crash avoidance potential of four passenger vehicle technologies[J]. Accid Anal Prev, 43(3):732-740.

[S15]. Flannagan, C. A., LeBlanc, D. J., Kiefer, R. J., Bogard, S., Leslie, A., Zagorski, C. T., ... Beck, C. S. (2018). Field study of light-vehicle crash avoidance systems: Automatic emergency braking and dynamic brake support (Report No. DOT HS 812 615). Washing-ton, DC: National Highway Traffic Safety Administration.

[S16]. Alkim, T.P., Bootsma, G., Hoogendoorn, S.P. (2007). Field operational test" the assisted driver". June In: In 2007 IEEE Intelligent Vehicles Symposium. IEEE. pp. 1198–1203.

[S17]. Lehmer, M., Miller, R., Rini, N., Orban, J., McMillan, N., Stark, G., Christiaen, A. (2007). Volvo Trucks Field Operational Test: Evaluation of Advanced Safety Systems for Heavy Trucks. US Department of Transportation National Highway Traffic Safety Administration.

[S18]. Cafiso, S., Di Graziano, A. (2012). Evaluation of the effectiveness of ADAS in reducing multi-vehicle collisions. Int. J. Heavy Veh. Syst. 19 (2), 188–206.

[S19]. Benmimoun M., Pütz A., Zlocki A., Eckstein L. (2013). euroFOT: Field Operational Test and Impact Assessment of Advanced Driver Assistance Systems: Final Results. In: SAE-China, FISITA (eds) Proceedings of the FISITA 2012 World Automotive Congress. Lecture Notes in Electrical Engineering, vol 197. Springer, Berlin, Heidelberg. https://doi.org/10.1007/978-3-642-33805-2_43

[S20]. Flannagan, C., & Leslie, A. (2020). Crash avoidance technology evaluation using real_x0002_world crash data (Report No. DOT HS 812 841). Washington, DC: National Highway Traffic Safety Administration

删除[H]: "
删除[H]: , 2012
删除[H]: '
删除[H]: , June 2009
删除[H]: (2017)
删除[H]:
删除[H]: (2015)
删除[H]: ,
删除[H]: , DOI:
删除[H]: "
删除[H]: , 2012
删除[H]: 2011,
删除[H]: , October
删除[H]: *
删除[H]: ,
删除[H]: ,
删除[H]: ,
删除[H]: , March

[S21]. Kusano, K., Gorman, T. I., Sherony, R., & Gabler, H. C. (2014). Potential occupant injury reduction in the US vehicle fleet for lane departure warning–equipped vehicles in single-vehicle crashes. Traffic injury prevention, 15(sup1), S157-S164. 10.1080/15389588.2014.922684

[S22]. Scanlon, J. M., Kusano, K. D., Sherony, R., & Gabler, H. C. (2015). Potential safety benefits of lane departure warning and prevention systems in the US vehicle fleet. In International Conference on Enhanced Safety of Vehicles (ESV).

[S23]. Luke E. Riexinger, Rini Sherony & Hampton C. Gabler. (2019). Residual road departure crashes after full deployment of LDW and LDP systems, Traffic Injury Prevention, 20:sup1, S177-S181, DOI: 10.1080/15389588.2019.1603375

[S24]. Hickman, J. S., Feng, G., Camden, M. C., Medina, A., Hanowski, R. J., & Mabry, E. (2013). Onboard Safety Systems Effectiveness Evaluation Final Report (No. FMCSA-RRT-12-012). United States. Federal Motor Carrier Safety Administration.

[S25]. Cicchino, J.B. (2018). Effects of lane departure warning on police-reported crash rates. J. Safety Res. 66, 61–70.

[S26]. Nodine, E., A. Lam, S. Stevens, M. Razo and W. Najm. (2011). Integrated vehicle-based safety systems (IVBSS) light vehicle field operational test independent evaluation. Washington, D.C., National Highway Traffic Safety Administration.

[S27]. Simon Sternlund, Johan Strandroth, Matteo Rizzi, Anders Lie & Claes Tingvall. (2017). The effectiveness of lane departure warning systems—A reduction in real-world passenger car injury crashes, Traffic Injury Prevention, 18:2, 225-229, DOI: 10.1080/15389588.2016.1230672

[S28]. Spicer, R., Vahabaghaie, A., Bahouth, G., Drees, L., Martinez von Bülow, R., & Baur, P. (2018). Field effectiveness evaluation of advanced driver assistance systems. Traffic injury prevention, 19(sup2), S91-S95.

[S29]. Irene Isaksson-Hellman, Magdalena Lindman. (2018). An evaluation of the real-world safety effect of a lane change driver support system and characteristics of lane change crashes based on insurance claims data. Traffic Injury Prevention 19:sup1, pages S104-S111.

[S30]. Jessica B. Cicchino. (2018). Effects of blind spot monitoring systems on police-reported lane-change crashes, Traffic Injury Prevention, 19:6, 615-622

[S31]. Schaudt, W. A., Bowman, D. S., Darrell, R. J., Olson, R. L., Marinik, A., Soccolich, S., ... & Rice, J. C. (2014). Federal Motor Carrier Safety Administration's Advanced System Testing Utilizing a Data Acquisition System on the Highways (FAST DASH): Safety Technology Evaluation Project# 1 Blindspot Warning (No. FMCSA-RRT-13-008). United States. Federal Motor Carrier Safety Administration.

设置格式[H]: 字体: 10 磅, 字体颜色: 自动设置, （中文）

删除[H]: Kristofer Kusano, Thomas I. Gorman, Rini Sherony & Hampton C. Gabler (2014) Potential Occupant Injury Reduction in the U.S. Vehicle Fleet for Lane Departure Warning–Equipped Vehicles in Single-Vehicle Crashes, Traffic Injury Prevention, 15:sup1, S157-S164, DOI:

删除[H]:

设置格式[H]: 字体: 10 磅, 字体颜色: 自动设置, （中文）

删除[H]: J.M. Scanlon, K.D. Kusano, R. Sherony, H.C. Gabler. Potential Safety Benefits of Lane Departure Warning and Prevention Systems in the US Vehicle Fleet, NHTSA Proceedings (2015)

设置格式[H]: 字体: 10 磅, 字体颜色: 自动设置, （中文）

删除[H]: Hickman, J. S., Guo, F., Camden, M. C., Medina, A., Hanowski, R. J., Mabry, E. "On board Safety System Effectiveness Evaluation Final Report." Report No. FMCSA-RRT-12-012. Washington, D.C.: Federal Motor Carrier Safety Administration, 2013

删除[H]: ,

设置格式[H]: 字体: 10 磅, 字体颜色: 自动设置, （中文）

删除[H]: Rebecca, Spicer, Amin, et al. Field effectiveness evaluation of advanced driver assistance systems.[J]. Traffic Injury Prevention, 2018

设置格式[H]: 字体: 10 磅, 字体颜色: 自动设置, （中文）

删除[H]: Schaudt, W.A., Bowman, D.S., Hanowski, R.J., Olson, R.L., Marinik, A., Soccolich, S.,et al., 2014. Federal Motor Carrier Safety Administration's Advanced System Testing Utilizing a Data Acquisition System on the Highways (FAST DASH): Safety Technology Evaluation Project# 1 Blindspot Warning (No. FMCSA-RRT-13-008)

[S32]. Chang, J. (2016). Summary of NHTSA heavy-vehicle vehicle-to-vehicle safety communications research. (Report No. DOT HS 812 300). Washington, DC: National Highway Traffic Safety Administration

[S33]. Harding, J., Powell, G., R., Yoon, R., Fikentscher, J., Doyle, C., Sade, D., Lukuc, M., Simons, J., & Wang, J. (2014). Vehicle-to-vehicle communications: Readiness of V2V technology for application. (Report No. DOT HS 812 014). Washington, DC: National Highway Traffic Safety Administration

[S34]. Chen, H., Cao, L., Logan, D.B. (2011). Investigation into the effect of an intersection crash warning system on driving performance in a simulator. Traffic Inj. Prev. 12 (5), 529–537.

[S35]. NHTSA. (2016). Preliminary Regulatory Impact Analysis: FMVSS No. 150, Vehicle-to-Vehicle Communication Technology for Light Vehicles. NHTSA, 2. Rep. DOT HS 812 359, 2016

[S36]. John M. Scanlon, Rini Sherony & Hampton C. Gabler. (2017). Injury mitigation estimates for an intersection driver assistance system in straight crossing path crashes in the United States, Traffic Injury Prevention, 18:sup1, S9-S17, DOI: 10.1080/15389588.2017.1300257

[S37]. John M. Scanlon, Rini Sherony, Hampton C. Gabler. (2016). Preliminary potential crash prevention estimates for an Intersection Advanced Driver Assistance System in straight crossing path crashes. 2016 IEEE Intelligent Vehicles Symposium (IV), pages 1135-1140.

[S38]. Ulrich Sander, Nils Lubbe. (2018). Market penetration of intersection AEB: Characterizing avoided and residual straight crossing path accidents. Accident Analysis & Prevention. Volume 115, Pages 178-188

[S39]. Bareiss, M., Scanlon, J., Sherony, R., & Gabler, H. C. (2019). Crash and injury prevention estimates for intersection driver assistance systems in left turn across path/opposite direction crashes in the United States. Traffic injury prevention, 20(sup1), S133-S138.

[S40]. The Ministry of Public Security of the People’s Republic of China. (2020). Annual Report on Road Traffic Accidents of the People's Republic of China

删除[H]: , July

删除[H]: , August

删除[H]: ,

设置格式[H]: 字体: 10 磅, 字体颜色: 自动设置, （中文）

删除[H]: NHTSA, “Preliminary regulatory impact analysis: FMVSS No. 150, vehicle-to-vehicle communication technology for light vehicles,” U.S. Dept. Transp., NHTSA, Washington, DC, USA, Tech.

删除[H]: June 2018,

设置格式[H]: 字体: 10 磅, 字体颜色: 自动设置, （中文）

删除[H]: Max Bareiss,John Scanlon,Rini Sherony &Hampton C. Gabler. 2019. Crash and injury prevention estimates for intersection driver assistance systems in left turn across path/opposite direction crashes in the United States. traffic injury prevention

删除[H]: (2019), July 20

删除[H]: 20
